# Oral Health-Related Quality of Life in Chronic Liver Failure Patients Measured by OHIP-14 and GOHAI

**DOI:** 10.1155/2020/8835824

**Published:** 2020-12-26

**Authors:** Maryam Zahed, Mohammad Ali Ranjbar, Azita Azad

**Affiliations:** ^1^Oral and Dental Disease Research Center, Department of Oral and Maxillofacial Medicine, School of Dentistry, Shiraz University of Medical Sciences, Shiraz, Iran; ^2^Student Research Committee, School of Dentistry, Shiraz University of Medical Sciences, Shiraz, Iran

## Abstract

**Background:**

Oro-dental diseases are prevalent in chronic liver failure (CLF) patients. The aim of this study was to evaluate the quality of life associated with oral health in candidates for liver transplant surgery.

**Materials and Methods:**

The demographic information of 105 end-stage liver cirrhotic patients was collected. All patients were ordered a panoramic view for pretransplant dental evaluation. The DMFT (decayed-missing-filled tooth) index was calculated for dental examination. The model for end-stage liver disease (MELD) was used for the severity of liver disease. The OHIP-14 (Oral Health Impact Profile) questionnaire and GOHAI (Geriatric Oral Health Assessment Index) questionnaire were applied to evaluate the impact of oral disease on the quality of life.

**Results:**

A total of 79 patients thoroughly completed the questionnaires; 79.7% were male, 32.9% were over 50, and 25.3% were less than 30 years old. Further, 12.7% smoked, 2.5% were illiterate, 64.6% had not finished school, and 10.1% had university degrees. Almost half of the cirrhotic patients were suffering from the disease for more than 3 years. Most complaints reported by the patients as “very often” were becoming self-conscious (13.9%) and being uncomfortable when eating any foods (13.9%) followed by feeling tense (12.8%). There was no significant difference between gender, smoking, age, and MELD score based on quality of life (OHIP and GOHAI) (*P* > 0.05). The level of education (*P* = 0.020), duration of disease (*P* = 0.017), and DMFT index (*P* = 0.039) had a significant impact on oral health-related quality of life in CLF patients. An inverse relationship was seen between the DMFT index and the quality of life.

**Conclusion:**

Oral health has a high impact on the quality of life of cirrhotic patients. The psychological dimension of oral health is the most debilitating factor affecting the quality of life. This shows the importance of professional oral care, oral health, and self-care education in this group of patients.

## 1. Introduction

Cirrhosis or chronic liver failure (CLF) is the liver end-stage disease that is manifested by damage to the tissue and structures of this organ. The only treatment at this stage remains to be liver transplantation surgery [1]. The consequence of this disease can affect all body structures including the oral mucosa, jawbones, and teeth [2, 3].

The period of illness before transplantation is associated with increased physical, psychological, and social stress. This stress is due to complications such as impaired sexual function, change of appearance, pain, limited social interactions, and reduced job satisfaction. These complications increase emotional stress, anxiety, and depression. They also reduce adaptation and self-confidence, and above all, they decrease self-care behaviors, especially in the field of oral and dental health [3–7]. One of the important outcomes of oral diseases and poor oral health is the psychological and social impact on an individual's life [8–10].

Studies reveal that saliva production is reduced in CLF patients. This results in an increase in the rate of dental caries and opportunistic infections such as fungal-related lesions [3]. It is ascertained that oral infections affect the success of future transplant surgery. Thus, the importance of oral health in CLF patients is further elucidated [2, 11].

Health-related quality of life (HRQOL) of CLF patients is an important issue that has been addressed in many previous studies [4, 5]. HRQOL means a person's perception and satisfaction of his physical and mental characteristics, from which he is able to perform his daily activities. This definition includes physical, mental, psychological, and social health as well as the ability to perform satisfactory daily actions [12].

Nowadays, with the importance of a patient-centered approach in clinical decisions, attention to oral health-related quality of life (OHRQOL) plays a special role in patient care [9]. OHRQOL measures the effect of various oral diseases, as well as the impact of preventive programs and dental treatment interventions on the quality of life of individuals [13]. Tooth decay and periodontal problems cause physical, functional, and biological complications. They also affect the economic, social, and psychological dimensions of patients [8, 14].

Numerous instruments have been proposed for measuring OHRQOL. Oral Health Impact Profile (OHIP-14) is a 14-item questionnaire that addresses the limitations, disabilities, and discomforts related to oral disease. Higher scores indicate a greater problem in oral health [13]. Furthermore, the GOHAI (Geriatric Oral Health Assessment Index) questionnaire is also an assessment tool for examining the relationship between oral diseases and quality of life in the elderly. This tool also addresses three main dimensions: (1) physical function, (2) psychosocial function, and (3) pain or discomfort [15].

Accordingly, considering the importance of oral health in liver transplant success and its consequent impact on the quality of life of these patients, we decided to design this study to assess the effects of oral health status and disease severity on OHRQOL of candidates for liver transplant surgery.

## 2. Patients and Methods

### 2.1. Study Group

Liver transplantation in Iran is centralized in Nemazee Hospital, Shiraz, southern Iran. This cross-sectional study enrolled eligible candidates for liver transplantation who were referred to Imam Reza Dental Clinic in Shiraz, Iran, for pretransplant dental evaluation in summer 2019. The inclusion criteria were adult patients above 18 with the initial diagnosis of chronic liver failure confirmed by pathologic evaluation and clinical examination by a member of the transplant team. Patients with a history of head and neck trauma, major systemic problem causing changes in pain sensation, fibromyalgia, edentulous subjects, any systemic disease affecting the dentition and oral structures (such as diabetes mellitus, oral lichen planus, lichenoid reactions, pemphigus vulgaris, AIDS, history of head and neck radiation, Sjogren's syndrome, and Behҫet's disease), use of any medication with known effects on the oral cavity (antidepressants and tranquilizers), and individuals who were not willing to participate in the study were excluded. Further, patients were initially examined, and if any sign of oral dryness and dental anomalies were detected, they were also excluded from the study.

### 2.2. Ethical Considerations

Written informed consent was obtained from all patients who participated in the study. All information about individuals was coded and kept confidential. This study was approved by the Ethics Committee of Shiraz University of Medical Sciences (IR.SUMS.DENTAL.REC.1398.071).

### 2.3. Data Collection Procedure

#### 2.3.1. Sociodemographic and Disease Characteristics

Age, gender, level of education, smoking status, and duration of disease were recorded from the patients' medical records and direct interviews. Chronic liver disease patients were categorized according to the severity of liver disease using the model for end-stage liver disease (MELD) scoring system. The MELD score in the present study was calculated with the blood creatinine, bilirubin, and INR (international normalized ratio) values recorded at the time of listing for liver transplant surgery by the transplant team. MELD scores were divided into three groups: low (MELD < 10), medium (MELD 11–18), and high (MELD 19–40).

#### 2.3.2. Dental Evaluation

Panoramic radiography was performed for all patients, along with a thorough dental examination by an oral and maxillofacial medicine specialist to record the DMFT (decayed-missing-filled tooth) index. Note that a panoramic view is ordered for all patients (dentate and edentulous) prior to transplant surgery to rule out any source of dental and bone pathologies and infections in the maxillary plus mandibular region.

#### 2.3.3. OHRQOL Assessment

We used the OHIP-14 questionnaire and the GOHAI (Geriatric Oral Health Assessment Index) questionnaire to assess OHRQOL. The OHIP-14 questionnaire consists of 14 five-choice questions. The scores in this questionnaire are coded as follows: 5 = very often, 4 = fairly often, 3 = occasionally, 2 = hardly ever, and 1 = never. This questionnaire covers 7 aspects of OHRQOL including functional limitations, physical pain, mental distress, physical disability, mental disability, social disability, and handicap. In this questionnaire, all questions have a negative impression, so the score of all questions with good oral conditions is inverse. Thus, higher scores (range 14-70) would indicate a lower level of OHRQOL. The validity of this questionnaire has been confirmed in previous studies, and its Persian format is available [16]. GOHAI (Geriatric Oral Health Assessment Index) addresses 3 dimensions of quality of life: physical (physical), social and psychological (psychosocial), and pain and discomfort (pain and discomfort). This questionnaire has twelve items previously used for the elderly, but they are now available for all ages. The same scoring system as the OHIP-14 was used with higher scores (range 12-60) indicating a lower level of OHROQL. The validity of this questionnaire has been confirmed in previous studies, and its Persian format is available [17]. Illiterate patients were interviewed for both questionnaires.

### 2.4. Data Processing and Analysis

Finally, statistical data were collected with SPSS software version 24 (SPSS Inc., Chicago, IL, USA) used for data analysis. Descriptive statistics including frequency and mean levels were used to describe the data. The Spearman correlation coefficient was also employed to investigate the relationship between the DMFT indices and the quality of life. One-way ANOVA was utilized to compare the groups given the normality of the variables. A significance level of less than 5% was considered significant.

## 3. Results

### 3.1. Sociodemographic Characteristics

**Figure 1:**
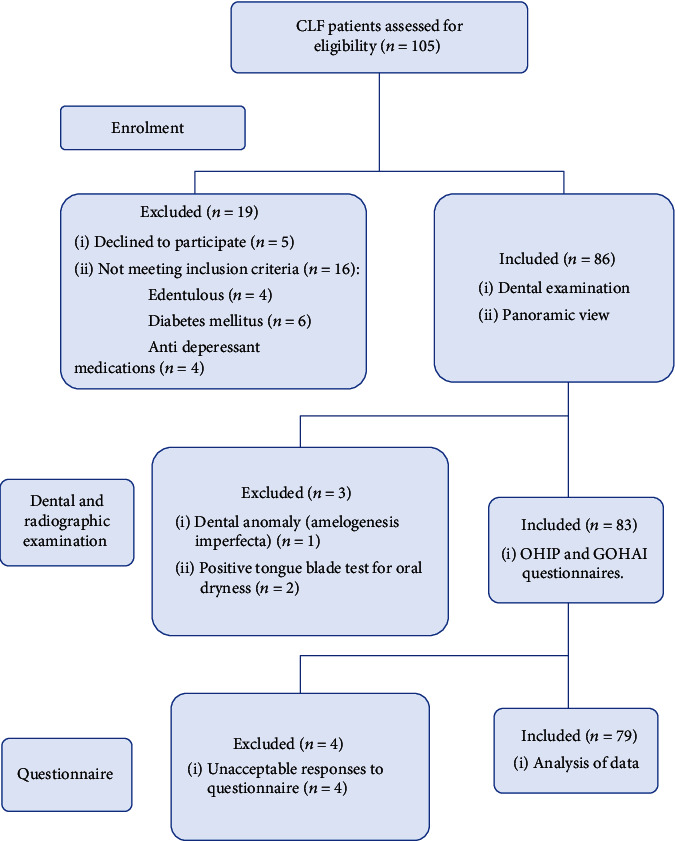
Flow chart of participants included in the study.

A total of 79 completed questionnaires were acceptable to be enrolled in the study, as seen in Figure 1. The distribution of chronic liver failure patients by gender, age, education, duration of disease, and smoking status is listed in Table 1. Based on this table, men over 50 are more likely to be candidates for liver transplant surgery. Furthermore, almost 65% of the patients had only middle school education and 87% were nonsmokers.

### 3.2. OHRQOL Characteristics

The mean score for the OHIP-14 questionnaire in CLF patients was 25.00 ± 10.02. This score was 23.54 ± 8.27 for the GOHAI questionnaire. The responses to the OHIP-14 items are represented in Table 2. As shown, the distribution of the patients' responses is almost uniform to all the OHIP-14 items. The mean scores for each question ranged between 1.25 for totally unable to function and 2.64 for having been self-conscious because of their teeth, mouth, or partial dentures. The major complaint reported by the patients as “never” was related to having trouble pronouncing any words (84.8%), followed by totally unable to function (83.5%) and unsatisfactory diet (83.5%). The major complaints reported by the patients as “very often” were becoming self-conscious (13.9%) and being uncomfortable when eating any foods (13.9%), followed by feeling tense (12.8%). The GOHAI distribution of answers was almost similar to the OHIP-14 questionnaire distribution.

### 3.3. The Effects of Different Variables on OHRQOL

The results of this study revealed that there is no significant difference between the mean quality of life based on gender, age, smoking, and MELD score according to both the OHIP-14 and GOHAI questionnaires (*P* > 0.05). This relationship, however, was significant for the level of education by the GOHAI questionnaire (*P* = 0.020). Disease duration was also significantly related to OHRQOL in this group (*P* = 0.017).

**Figure 2:**
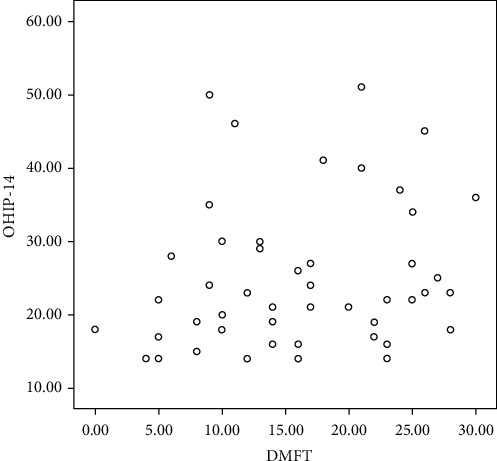
Correlation of OHIP-14 with the DMFT index in CLF patients.

The results of the Pearson correlation for the assessment of the DMFT index and OHRQOL revealed a significant relationship between the two variables based on the OHIP-14 questionnaire. This means that as the DMFT index in cirrhotic patients increased, so did the mean score of the OHIP-14 questionnaire, showing the reduction of OHRQOL (*P* = 0.039) (Table 3 and Figure 2).

## 4. Discussion

The results of this study revealed that most of the end-stage liver cirrhotic patients were male, were over 50 years old, and had not finished high school. Nearly half had been suffering from liver disease for more than 3 years. Interestingly, the results elucidated that oral and dental complications are effective in reducing the quality of life in patients suffering from CLF. This is also true for the level of education and duration of sickness, which can both significantly affect the OHRQOL in this group of patients. Furthermore, the major complaints reported by the patients were becoming self-conscious because of their teeth, mouth, or dentures.

There are many factors that initiate and accelerate the rate of dental caries: (1) genetic factors such as the immune system, saliva concentration, and composition, as well as teeth anatomy plus its hard tissue quality; and (2) environmental factors such as nutrition, oral hygiene, socioeconomic level, and mental status [18]. According to the mentioned factors, it should be expected that in a patient who has reached the end-stage of chronic disease, the immune system, nutritional status, and oral hygiene are highly affected, and therefore, the incidence of dental cavities, periodontal problems, and DMFT rate increased [19]. As previously shown, end-stage liver disease patients are prone to oral infections compared to healthy individuals [3]. Furthermore, periapical lesions of teeth, which are the result of chronic dental infections, are more prevalent in this group [20, 21]. Hence, the existence of oral and dental problems causes pain and discomfort, disturbs the patient's nutrition, and affects appearance and esthetics, all of which can influence their quality of life [3, 20].

We found that with an increase in the DMFT index, the quality of life was significantly reduced in CLF patients. Contrary to the results of this study, in a study conducted by Schmalz et al., OHRQOL was evaluated before and after liver transplant surgery. This study indicated a reduced OHRQOL compared to healthy individuals but not related to oral complications in this group of patients. They concluded that further studies with a larger population are warranted to confirm this matter [22]. Likewise, in the study of Mohammadzadeh et al., the components of the DMFT index by D, M, and F did not show a difference in terms of quality of life among the patients [14]. However, this study did not consider patients with systemic disease. A study with a similar result to the present study showed that factors such as tooth decay and bad breath in patients with oral complications can reduce the quality of life, as well as physiological and mental ability [23]. This is similar to our study which shows that the psychological aspects of oral health are the most debilitating factor in CLF patients. It is also proved that the reduced rate of caries and improved oral hygiene augment the quality of life [8].

Additionally, regarding the psychological aspect of oral health, our results are similar to other studies which found that becoming self-conscious claimed the largest score for answering “very often” [23]. Prior to the transplant procedure, a multidisciplinary evaluation is performed to assess the patient's suitability for this surgery. In this evaluation, the presence of psychological factors that could compromise the patient or graft survival must be ruled out. This highlights the importance of psychological health in CLF patients [24]. Our findings support the need for oral health education and oral hygiene instructions to reduce the psychological burden of oral complications in CLF patients.

The MELD score is used in CLF patients to show the severity of the disease and predict the overall prognosis. Many countries use it for the allocation of patients for liver transplant surgery. We did not find any relations between the MELD score and the OHRQOL. This is similar to the results of other studies which did not find any significant relations regarding MELD scores and oral health status [3, 21]. However, there are studies that did find a relationship between severity of liver disease (MELD score) and oral health status [24]. Note that neither of these studies evaluated OHRQOL.

In relation to patients suffering a systemic disease and quality of life, a study conducted by Cervino et al. elucidated that oral complications affect the quality of life in diabetic individuals [25]. Helenius-Hietala et al. and Zwiech and Bruzda-Zwiech in two separate studies examined the effect of oral and dental infections on the quality of life and course of disease of two groups of kidney and liver patients. Both studies showed that oral infections had a negative effect on disease improvement as well as on the quality of life [2, 11]. It is important to note that the presence of oral lesions in cirrhotic patients as the focus of infection can affect the prognosis of the future transplant procedure and would cause serious problems for patients.

In regard to the level of education, we found that chronic liver failure patients who had finished high school and patients who had bachelor's degrees and above had the lowest level of OHRQOL according to the GOHAI questionnaire. In general, it is stated that low levels of education have a negative impact on oral health-related quality of life [26, 27]. Other researchers have also shown that with higher education, patients' awareness of chronic diseases and their ability to cope with its complications increase, and hence, the quality of life will improve [28]. These findings are contrary to the results of the present study. We suggest that although educated patients are more aware of the complications and problems of the disease which in some cases helps improve their condition, the expectations of such people from life and its quality are far higher. Thus, in the case of a chronic disease with no permanent cure, such patients are driven away from their desired life expectations, which in turn directly reduces their quality of life. Nevertheless, note that the distribution of the education level was not homogenous in our study. We propose a larger sample size with sufficient participants in each group in future studies.

The present study showed that there is a significant difference between the duration of illness and the quality of life (OHIP). In a study conducted in 2017 by Busija et al., they also found a significant relationship between disease duration and quality of life of patients, which was also related to their age and the significant effect of disease duration on quality of life [29]. Our results revealed that patients who have been ill for less than a year or have had the disease for more than three years report a higher quality of life. This may be because in the first year patients are not yet fully aware of their disease and its complications. Further, they are not yet seriously involved in the side effects of the disease and medications, and there are still minor oral and dental problems. However, during the second year, patients are more entangled with the complications of the disease, and they are more driven away from social and individual activities, all inducing more anxiety, stress, sadness, and fear. On the other hand, patients whose disease duration has been extended are somehow more familiar with the treatment course of their disease and have become more adaptable with the complications. In other words, they have become more accustomed to the disease and have accepted it.

Since CLF patients suffer from a chronic disease and its complications, encouraging the patients to participate in the study was somehow difficult. So, further studies with larger sample sizes are suggested. It is also suggested that, in future studies, the effects of oral hygiene habits and nutritional status of the patients should be considered on OHRQOL.

## 5. Conclusion

Finally, we can conclude that the quality of life related to oral health in candidates for liver transplant surgery is affected by their education and the duration of the disease as well as the DMFT index. The psychological dimensions of oral health are the most debilitating aspect. This can affect the outcomes of the transplant procedure. Thus, we support the importance of oral hygiene instructions with emphasis on self-care in end-stage liver cirrhotic patients to reduce its psychological aspect and its impact on the success of the transplant surgery.

## Figures and Tables

**Table 1:** OHRQOL according to OHIP-14 and GOHAI in CLF patients, based on gender, age, level of education, smoking, and disease duration.

Chronic liver failure patients		Number	Percentage	Mean OHIP-14	Mean GOHAI
Gender	Male	63	79.7	24.55 ± 9.15	23.93 ± 7.38
	Female	16	20.3	26.75 ± 13.08	22.0 ± 11.28
	*P* value (ANOVA)			0.438	0.407

Age	Below 30	20	25.3	24.65 ± 11.95	21.10 ± 8.24
	31-40	17	21.5	28.58 ± 3.12	26.82 ± 11.80
	41-50	16	20.3	24.6 ± 1.97	24.81 ± 8.03
	Above 50	26	32.9	23.50 ± 1.37	22.50 ± 4.45
	*P* value (ANOVA)			0.409	0.157

Education	Illiterate	2	2.5	18.50 ± 0.70	19.00 ± 7.07
	Middle school	51	64.6	25.25 ± 9.49	22.80 ± 7.39
	High school diploma	11	13.9	20.09 ± 7.48	20.09 ± 4.39
	Associate's degree	7	8.9	23.28 ± 12.12	26.57 ± 12.81
	Bachelor's degree and above	8	10.1	33.25 ± 11.75	31.50 ± 9.33
	*P* value (ANOVA)			0.053	0.020^∗^

Smoking	Yes	10	12.7	25.10 ± 9.73	24.20 ± 8.98
	No	69	87.3	24.98 ± 10.13	23.44 ± 8.23
	*P* value (ANOVA)			0.973	0.791

Disease duration	Less than a year	20	25.3	20.90 ± 7.67	22.75 ± 7.86
	1-2 years	13	16.5	30.76 ± 10.98	28.46 ± 14.34
	2-3 years	8	10.1	29.87 ± 4.02	23.25 ± 6.08
	More than 3 years	38	48.1	24.15 ± 7.95	22.34 ± 8.91
	*P* value (ANOVA)			0.017^∗^	0.132

MELD	Low	14	17.72	26.44 ± 12.18	23.47 ± 10.38
	Medium	35	44.30	23.33 ± 8.27	23.03 ± 6.77
	High	30	37.97	25.06 ± 7.54	24.73 ± 5.48
	*P* value (ANOVA)			0.470	0.812

Total		79	100	25.00 ± 10.02	23.54 ± 8.27

MELD = model for end-stage liver disease. ^∗^*P* value < 0.05 was considered significant.

**Table 2:** The mean and percentage of answers to the Oral Health Impact Profile (OHIP-14) questionnaire.

Dimension	Variables	1 (%)	2 (%)	3 (%)	4 (%)	5 (%)	Mean score
Functional limitation	Have you had trouble pronouncing any words because of problems with your teeth, mouth, or dentures?	84.8	1.3	10.1	3.8	—	1.32
	Have you felt that your sense of taste has worsened because of problems with your teeth, mouth, or dentures?	75.9	3.8	12.7	—	7.6	1.59

Physical pain	Have you had painful aching in your mouth?	45.6	8.9	26.6	12.7	6.3	2.26
	Have you found it uncomfortable to eat any foods because of problems with your teeth, mouth, or dentures?	55.7	2.5	24.1	3.8	13.9	2.17

Psychological discomfort	Have you been self-conscious because of your teeth, mouth, or dentures?	55.7	2.5	24.1	3.8	13.9	2.64
	Have you felt tense because of problems with your teeth, mouth, or dentures?	62.8	10.3	6.4	7.7	12.8	1.96

Physical disability	Has your diet been unsatisfactory because of problems with your teeth, mouth, or dentures?	83.5	3.8	5.1	7.6	—	1.37
	Have you had to interrupt meals because of problems with your teeth, mouth, or dentures?	64.6	11.4	11.4	11.4	1.3	1.73

Psychological disability	Have you found it difficult to relax because of problems with your teeth, mouth, or dentures?	53.2	15.2	24.1	3.8	3.8	1.89
	Have you been a bit embarrassed because of problems with your teeth, mouth, or dentures?	62.0	5.1	16.5	7.6	8.9	1.96

Social disability	Have you been a bit irritable with other people because of problems with your teeth, mouth, or dentures?	65.4	12.8	14.1	2.6	5.1	1.68
	Have you had difficulty doing your usual jobs because of problems with your teeth, mouth, or dentures?	72.2	12.7	10.1	3.8	1.3	1.49

Handicap	Have you felt that life in general was less satisfying because of problems with your teeth, mouth, or dentures?	69.6	10.1	5.1	11.4	3.8	1.69
	Have you been totally unable to function because of problems with your teeth, mouth, or dentures?	83.5	10.1	3.8	2.5	—	1.25
Total							25.00

Never (=1), hardly ever (=2), occasionally (=3), fairly often (=4), and very often (=5).

**Table 3:** OHRQOL according to the DMFT index in CLF patients.

DMFT	Correlation coefficient	*P* value
OHIP-14	0.244	0.039^∗^
GOHAI	0.110	0.359

^∗^ *P* value < 0.05 was considered significant.

## Data Availability

Other data used to support the findings of this study are available upon request from the corresponding author.
